# Chronic oral LPS administration does not increase inflammation or induce metabolic dysregulation in mice fed a western-style diet

**DOI:** 10.3389/fnut.2024.1376493

**Published:** 2024-07-15

**Authors:** Silje Harvei, Vemund Skogen, Bjørg Egelandsdal, Signe Birkeland, Jan Erik Paulsen, Harald Carlsen

**Affiliations:** ^1^Faculty of Chemistry, Biotechnology and Food Science, Norwegian University of Life Sciences, As, Norway; ^2^Faculty of Veterinary Medicine, Norwegian University of Life Sciences, As, Norway

**Keywords:** obesity, LPS, intestinal permeability, low-grade inflammation, NF-κB activation

## Abstract

**Introduction:**

Lipopolysaccharides (LPS) present in the intestine are suggested to enter the bloodstream after consumption of high-fat diets and cause systemic inflammation and metabolic dysregulation through a process named “metabolic endotoxemia.” This study aimed to determine the role of orally administered LPS to mice in the early stage of chronic low-grade inflammation induced by diet.

**Methods:**

We supplemented the drinking water with *E. coli* derived LPS to mice fed either high-fat Western-style diet (WSD) or standard chow (SC) for 7 weeks (*n* = 16–17). Body weight was recorded weekly. Systemic inflammatory status was assessed by *in vivo* imaging of NF-κB activity at different time points, and glucose dysregulation was assessed by insulin sensitivity test and glucose tolerance test near the end of the study. Systemic LPS exposure was estimated indirectly via quantification of LPS-binding protein (LBP) and antibodies against LPS in plasma, and directly using an LPS-sensitive cell reporter assay.

**Results and discussion:**

Our results demonstrate that weight development and glucose regulation are not affected by LPS. We observed a transient LPS dependent upregulation of NF-κB activity in the liver region in both diet groups, a response that disappeared within the first week of LPS administration and remained low during the rest of the study. However, WSD fed mice had overall a higher NF-κB activity compared to SC fed mice at all time points independent of LPS administration. Our findings indicate that orally administered LPS has limited to no impact on systemic inflammation and metabolic dysregulation in mice fed a high-fat western diet and we question the capability of intestinally derived LPS to initiate systemic inflammation through a healthy and uncompromised intestine, even when exposed to a high-fat diet.

## Introduction

Lipopolysaccharides (LPS) are the major components of the outer membrane of Gram-negative bacteria, naturally present in the human intestine as part of the microflora. LPS consists of a polysaccharide and a lipid component named lipid A. The host innate immune system recognizes LPS (lipid A) to mount a response against potentially pathogenic intruders. The recognition typically involves an initial binding to the LPS-binding protein (LBP), which delivers LPS to a complex consisting of CD14, MD-2 and Toll-like receptor 4 (TLR4; (1)). The LPS-TLR4 interaction initiates an inflammatory response characterized by increased levels of proinflammatory cytokines mediated by the transcriptional regulator nuclear factor-κB (NF-κB). This may lead to an acute local response or a systemic response depending on the LPS levels and where the LPS are found (2, 3). Minor increases of LPS in the circulatory system are proposed to induce low-grade inflammation, associated with metabolic dysregulation including insulin resistance and overweight (4–6).

LPS is seemingly detectable at very low concentrations in the circulation of healthy individuals, and it has been suggested that LPS can translocate through the intestinal wall and increase systemic LPS levels following ingestion of certain dietary patterns, leading to a phenomenon called “metabolic endotoxemia” (4). LPS translocation from the intestine into the blood may increase following ingestion of high fat, high caloric or high carbohydrate diets (6–8). Presently, dietary fat is the major dietary factor known to facilitate the translocation of LPS from the intestine (9–14) but seems to be augmented by diets high in sugar (15). Despite this being the common perception in the scientific community, the mechanisms connecting diet and elevated blood LPS with low-grade inflammation are still not well-understood. Of note, the original study demonstrating a role for LPS causing metabolic endotoxemia was based on systemic infusion of LPS using an implanted osmotic pump, thereby surpassing the physiological route for intestinal-derived LPS (4). In contrast, Dalby and coworkers demonstrated that diet-induced obesity is not dependent on TLR4 or the cluster of differentiation 14 (CD14) signaling (16), questioning the significance of LPS in causing or contributing to metabolic endotoxemia. Most of the studies supporting high-fat diet-induced metabolic endotoxemia are based on the experimental results of increased LPS in blood samples as determined by the Limulus amebocyte lysate (LAL) assay (4, 6, 17). The usefulness of the LAL assay is controversial as the test does not discriminate on the TLR4-stimulating properties of LPS. Detection of LPS from blood samples is regarded as difficult and susceptible to inaccuracies and is highly influenced by both inhibiting and enhancing factors (see reviews by Boutagy, McMillan (18) and Gnauck, Lentle and Kruger (19) for details). Furthermore, the same reviews note that the range of LPS blood concentrations in humans reported in the literature is highly variable, making it difficult to identify the quantifiable threshold between the normal and the inflammatory state.

The major source of circulating LPS is likely commensal bacteria residing in the gastrointestinal tract (GIT), which is a rich reservoir of Gram-negative bacteria (20). However, translocated LPS may also originate from the environment through the ingestion of common food items (21–23). Erridge and colleagues found that several common processed food items had significant amounts of TLR4-stimulants (21–23) suggesting that modern diets, characterized by processed- and pre-packaged foods, are associated with increased oral exposure to stimulants of TLR4, such as LPS. However, it has also been observed that different bacteria produce different types of LPS, some of which are less likely to elicit an inflammatory response through the classical LPS receptor, TLR4 (24, 25), corroborating with findings that concentrations of TLR-stimulants are low in healthy murine ileum (26).

To the best of our knowledge, few studies to date have explored systematically over several weeks whether LPS given through the oral route of mice can influence metabolic and inflammatory processes evoked by a high-fat diet. Given the higher tolerance of mice to LPS compared to humans, we administered a high yet physiologically relevant dose of LPS derived from *E. coli* through the drinking water to mice subjected to either a high-fat western-style diet (WSD) or a standard low-fat chow diet (SC). Our primary objective was to ensure that the mice faced a substantial LPS challenge and to examine whether LPS could induce systemic low-grade inflammation. Additionally, we hypothesized that the consumption of a high-fat western-style diet would accelerate the impact of LPS exposure on the development of low-grade inflammation by promoting the translocation of LPS into circulation.

Our findings indicate that intestinal LPS can indeed trigger a systemic inflammatory response; however, this effect is modest and transient. Over the long term, exposure to LPS did not influence metabolic dysregulation, as evidenced by normal body weight increase and glucose regulation. Consequently, we conclude that LPS is unlikely to play an active role in promoting metabolic dysfunction and chronic low-grade inflammation in a physiologically relevant setting, where LPS is administered in a manner that does not surpass the protective mechanisms associated with the intestinal barrier.

## Methods

### Animal experiments

Mice (C57BL/6JRj) were bred in-house by crossbreeding female wild-type (WT) with male heterozygous NF-κB-luciferase reporter mice (NF-κB-luc) (27). Female mice (C57BL/6JRj) for breeding were purchased from Janvier Labs (Le Genest-Saint-Isle, France). Both NF-κB-luc and WT mice were used in the experiments. All mice were housed in individually ventilated cages (Innocage; Innovive, San Diego, CA) supplied with hardwood chips as bedding in a temperature and humidity-controlled room (24°C ± 2, 50% RH ±5) on a 12 h light–dark cycle, with free access to food and water. Diets used in the experiments were standard chow (SC) (3.5 kcal/g; 7.5 E% fat [soy oil], 17.5 E% protein [whey powder and soy protein concentrate], 75 E% carbohydrates [soy and various grains], RM1, Special Diets Services, Essex, United Kingdom), or a Western-style diet (WSD) (Research Diets, New Brunswick, NJ) D12079B (4.5 kcal/g; 41 E% fat [milk fat and corn oil] 15 E% protein [casein], 44 E% carbohydrates [sucrose and corn starch/maltodextrin]). Detailed diet composition is provided in the Supplementary Tables S1, S2. Estimated daily water intake per animal was 4–6 mL. *Escherichia coli* O55:B5 LPS was from Sigma-Aldrich (St. Louis, MO; Cat# L2880).

#### Tolerance of oral LPS and detection of LPS in the small intestine

Nineteen female eight-month-old WT mice were divided into four groups, three experimental groups (*n* = 4/group) and one control group (*n* = 7). The three experimental groups were all supplemented with the same dose of LPS (in the drinking water 0.33 mg/mL; ~2 mg LPS/mouse/day), using three different exposure times (1, 4, or 8 days of LPS administration). The control was given water only. All mice were fed a standard chow diet until termination with subsequent sampling of intestinal content for LPS analysis. Body weights and general welfare (activity, body posture, self-grooming) were recorded during the LPS-administration.

#### Effect of long-term oral LPS and western-style diet on obesity and insulin resistance

Sixty-five male mice aged 22 weeks were first randomly divided into two dietary groups receiving either western-style diet (WSD) or low-fat standard chow (SC) diet for 2 weeks. After 2 weeks, half of the WSD fed mice received LPS supplemented in drinking water (0.33 mg/mL, WSD-LPS), and the rest were given water only (WSD-W). The same set up was chosen for the SC-fed mice; half of the SC-fed were given LPS in the drinking water (0.33 mg/mL, SC-LPS), whereas the rest were only given water (SC-W). These four groups (WSD-LPS, WSD-W, SC-LPS and SC-W; *n* = 16–17 in each group) where then followed for five additional weeks until termination at week 7. Assuming a water intake of 6 mL/day, each mouse in the LPS-groups was exposed to ~2 mg LPS/day (28). Each group consisted of eight WT mice and eight NF-κB-luc mice. Body weight was recorded once a week, and food consumption every 2–3 days. From our own experience, chow-pellets cause ~15% more food spillage (food crumbles collected from cage beddings) than western diet-pellets when measuring food intake from cage feeders. Thus, we subtracted 15% from calculated energy intake for the SC diet-groups. Intraperitoneal insulin tolerance test (IpITT) and oral glucose tolerance test (OGTT) were performed in non-anesthetized mice in weeks 5 and 6, respectively. Mice were euthanized in week 7 of the experiment, corresponding to 5 weeks after LPS initiation, and tissue and blood samples were collected from fed mice.

#### Detection of orally administered LPS in plasma

Fifteen female WT 6 month-old mice were divided into two experimental groups (LPS) with two different LPS doses: 0.1 (*n* = 5) or 1 mg/mL (*n* = 5) and a control group receiving regular tap water (*n* = 5) for 8 days. Mice were fed a standard chow diet, and they were not fasted before sampling or termination.

### Ethics statement

All animal experiments were performed according to the national guidelines for animal welfare, and the protocol was approved by the Norwegian Food Safety Authority (FOTS ID 15600). Animal welfare was monitored by daily assessment of water and food intake, stool and fur quality, activity level and behavior.

### Tissue and blood samples

Mice were anesthetized by intraperitoneal (i.p.) injection of a mixture (10 μL/g mouse) denoted ZRF cocktail (Zoletil Forte; Virbac, Carros, France, Rompun; Bayer, Oslo, Norway and Fentadon; Eurovet Animal Health, Bladel, The Netherlands) with the active substances Zolazepam (32 mg/kg), Tiletamin (32 mg/kg), Xylanine (4.5 mg/kg) and Fentanyl (26 μg/kg), followed by cardiac puncture and blood collection from the heart (0.5–1 mL). Mice were then euthanized by cervical dislocation before dissecting out the organs. Luminal content for LPS assessments in the small intestine were collected, beginning at the base of the stomach, from the proximal 2 cm of the small intestine, and the distal 2 cm of the small intestine, respectively for duodenum and ileum. The protocol for preparation of the luminal content samples is derived from (29). Briefly, samples were diluted 1:4 (weight:volume) in sterile phosphate-buffered saline (PBS) and centrifuged at 13,000 g for 20 min before decanting the supernatant and storage of samples at −80°C before continuing the protocol at day of analysis. Whole intestinal samples for the IAP enzyme assay were collected from the next proximal 3 cm of the duodenum and cut into 1 cm pieces. Samples were snap frozen and stored at −80°C until analysis. Plasma from cardiac blood collected in an EDTA-covered syringe with a 25G needle was prepared by centrifugation at 6,000 g, 4°C for 10 min and stored at −80°C for further analyses.

### *In vivo* imaging of NF-κB activity

Imaging of transgenic mice expressing luciferase was performed with an IVIS Lumina Series III (PerkinElmer, Waltham, MA). Before imaging, the fur of the abdomen was shaved, and the mice anesthetized with 2.5% isoflurane (IsoFlo Vet 100% Isoflurane, Zoetis, Kalamazoo, MI). Mice were imaged in a fed state at 10 min post luciferin injection (15 mg/mL solution, 150 mg/kg mouse; D-Luciferin, Biosynth, Basel, Switzerland) with 5 min exposure time.

### Intraperitoneal insulin tolerance test and oral glucose tolerance test

When testing for insulin resistance, mice were fasted for 4 h and i.p. injected insulin (0.1 U/mL solution, 0.75 U/kg mouse; human insulin, Sigma-Aldrich, Schnelldorf, Germany) was followed by glucose measurements. When testing for glucose tolerance, mice were fasted for 6 h and given an oral bolus of glucose (0.2 g/mL solution, 2 g/kg mouse; D-glucose, Sigma Aldrich). For both tests, blood glucose from tail vein blood samples were quantified (Accu Chek Aviva, Roche Diagnostics, Mannheim Germany) at 0, 15, 30, 60, 90, and 120 min post insulin or glucose administrations.

### Measurement of plasma LBP, TNFα, and IL-6

Plasma LBP levels were measured by mouse LBP enzyme-linked immunosorbent assay (ELISA) kit (Biometec GmbH, Greifswald, Germany) and with mouse IL-6/TNFα Quantikine ELISA kit (R&D Systems, Minneapolis, MN). Plasma samples were diluted and assayed according to the manufacturer’s instructions.

### Gene expression analysis by RT-qPCR

Total mRNA was extracted from RNAlater preserved ileal mucosa samples following the NucleoSpin RNA/Protein (Macherey-Nagel, Germany) protocol with some minor alterations. Samples were homogenized in RP1/BME mix with a 23 G needle; each centrifugation step were increased by 1–2 min at 11,000 or 13,000 × g; and RNA was eluted in 50 μL RNase-free H_2_O. For cDNA synthesis 200 ng of total mRNA was digested and reverse-transcribed with the iScript cDNA Synthesis kit (Bio-Rad Laboratories, CA, United States) according to the manufacturer’s instructions and used in Quantitative PCR on the LightCycler 480 system (Roche Diagnostics, Germany). The detection gene expression was performed using 5× HOT FIREPol EvaGreen qPCR Supermix (Solis BioDyne, Estonia). The following target genes were assessed: Tumor necrosis factor alpha (*Tnfa*) and interleukin 1 beta (*Il1b*). The housekeeping gene glyceraldehyde-3-phosphate dehydrogenase (*Gapdh*) was used as a reference, and relative mRNA expression for both target genes in each sample were calculated using the formula $R=\left({E_{\mathrm{target}}}^{\left(-Cq_{\mathrm{target}}\right)}\right)/\left({E_{\mathrm{reference}}}^{\left(-Cq_{\mathrm{reference}}\right)}\right)$ where *E* denotes the primer efficiency. Primer sequences for *Tnfa*, *Il1b*, and *Gapdh* can be found in Supplementary Table S3. The mRNA extracts from all 16 mice per group were analyzed individually.

### Measurement of duodenal IAP activity

IAP activity was measured with the SensoLyte p-nitrophenyl phosphatase (pNPP) Alkaline Phoshatase Kit (Anaspec, Fremont, CA) according to recommendations of the manufacturer. Briefly, duodenal tissue was homogenized in lysis buffer (400 μL/50 mg tissue), centrifuged at 10,000 g for 15 min at 4°C, before diluting the supernatant 1:800. Samples were incubated with pNPP reaction mixture for 20 min, and absorbance was read at 405 nm.

### Measurement of plasma flagellin and LPS-specific immunoglobulins

Flagellin- and LPS-specific IgG levels were quantified by indirect ELISA based on previously published protocols (30, 31). Briefly, microtiter plates were coated overnight at 4°C with purified *Escherichia coli* (*E. coli*) flagellin (100 ng/well, orb107725, VWR, United Kingdom) or *E. coli* LPS (2 μg/well; serotypes O55:B5 [L4524] and O128:B12 [L2887], Sigma-Aldrich, St.Louis, MO, USA) in 0.05M carbonate-bicarbonate buffer pH 9.6. Plates were washed with wash buffer (PBS + 0.05% Tween®20 (Sigma-Aldrich, St.Louis, MO, USA)) and blocked for 1 h at 4°C with blocking buffer (1X ELISA Assay Buffer, Invitrogen^™^, Vienna, Austria (flagellin and LPS O128:B12 plates); 1X Reagent Diluent, R&D Systems, Minneapolis, MN, USA, (LPS O55:B5 plate)). Plasma samples were diluted 1:200 in corresponding blocking buffer and then applied to wells after another wash followed by incubation at 37°C for 1 hour. After incubation and another wash, the wells were incubated with anti-mouse IgG coupled to horseradish peroxidase (1:1000; Southern Biotechnology, Birmingham, AL, USA) at 37°C for 1 hour. Plates were washed before tetramethylbenzidine substrate (Thermo Scientific, Rockford, IL, USA (flagellin and LPS O128:B12 plates); R&D Systems, Minneapolis, MN, USA, (LPS O55:B5 plate)) was added to well for five minutes at room temperature and stopped using sulfuric acid (0.16M for flagellin and LPS O128:B12 plates; 1M for LPS O55:B5 plate (R&D Systems, Minneapolis, MN, USA)). Total immunoglobulins were quantified immediately using a microaplate reader (SpectraMax® M2, Molecular Devices, California, USA). Absorbance was measured at 450 nm and 540 nm, and IgG-levels are reported as optical density (OD) units corrected for background value blanks.

### Quantification of TLR4-stimulants in plasma and small intestine

Biologically active LPS, i.e., LPS that induced TLR4 signaling, were determined in plasma, luminal duodenum, and ileum samples by employing a colorimetric cell-based reporter assay (HEK-Blue LPS Detection Kit 2, InvivoGen, San Diego, CA). Quantification was done according to the manufacturer’s instructions. In brief, diluted plasma and intestinal samples were added to human embryonic kidney (HEK-293) cells that express mTLR4 and an NF-κB-inducible secreted embryonic alkaline phosphatase reporter gene. After 18 h incubation, cell culture media was applied to QUANTI-Blue medium from said assay to measure alkaline phosphatase activity. Plates were read at a wavelength of 620 nm. The detection limit was 0.03 ng/mL according to the kit manufacturer.

### Statistical analysis

Statistical analyses were performed using Prism version 9.4 (GraphPad Software, San Diego, CA). The results were expressed as mean ± standard error of the mean. The significance of difference between two groups was evaluated using the two-tailed Student’s *t*-test. Differences in continuous parameters among multiple groups were calculated using the one-way analysis of variance (ANOVA) with *post hoc* Tukey’s multiple comparisons test. Two-way ANOVA was used to assess effects of LPS exposure (LPS or water) in the two diet groups (WSD or SC). Significant interactions between LPS exposure and diet in two-way ANOVA were followed by the assessment of simple main effects (effect of LPS or water within each diet groups and effect of diet within LPS or water exposure groups) with Tukey’s correction for multiple comparisons. The results were considered as statistically significant at *p* < 0.05.

## Results

### Exogenous LPS reached the small intestine and mice tolerated continuous oral LPS administration

**Figure 1:**
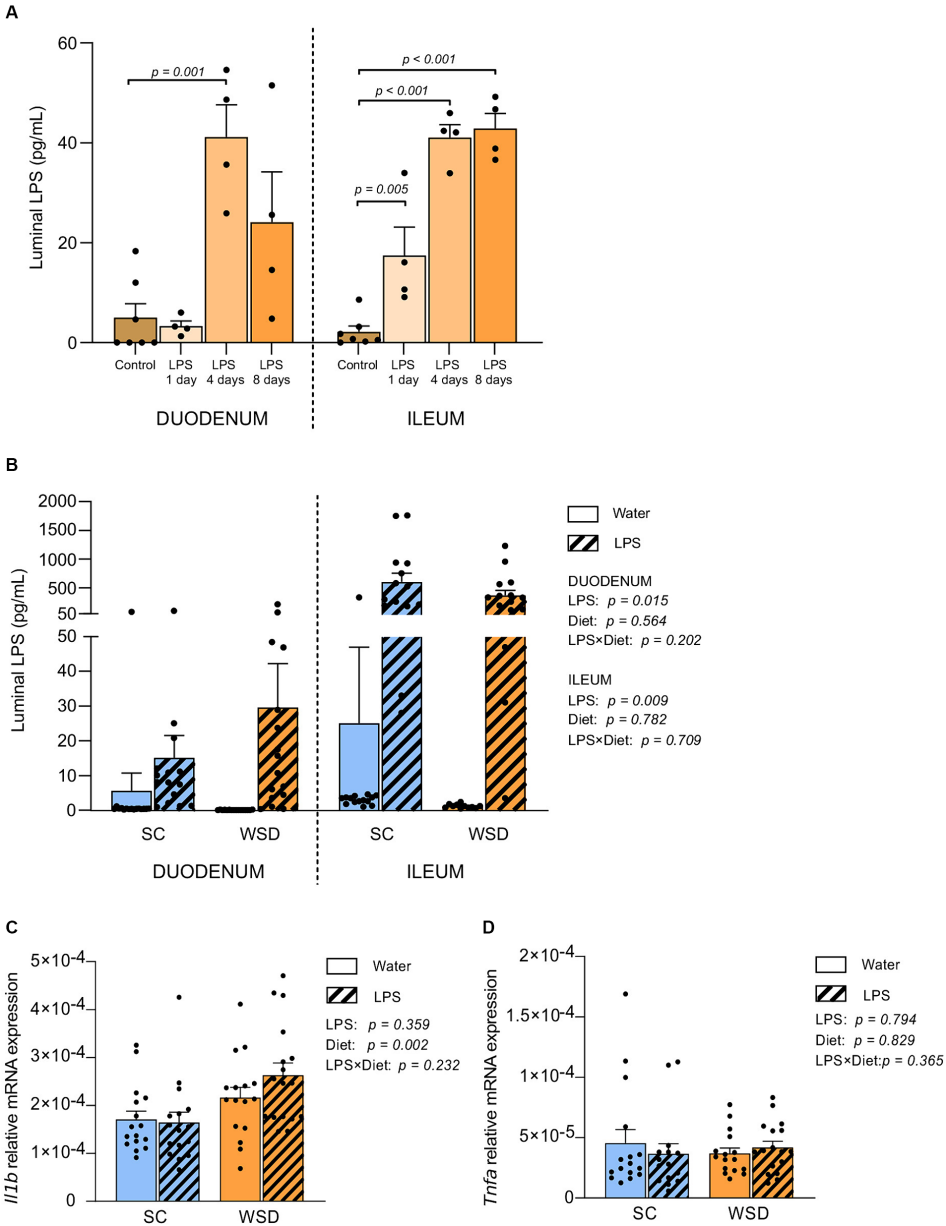
LPS content measured by the HEK-Blue^™^ TLR4 assay, and gene expression in the small intestine. **(A)** Duodenal and ileal LPS levels in luminal content from SC fed mice given LPS (0.33 mg/mL, estimated daily intake of 2 mg LPS) through drinking water for 1, 4 or 8 days. *N* = 4–7 per group. One-way ANOVA compared to the mean of control group (non-treated mice). **(B)** Duodenal and ileal LPS levels in mice fed WSD or SC diet for 7 weeks, and 35 days of oral LPS administration (0.33 mg/mL, estimated daily intake of 2 mg LPS), *n* = 11–17. Relative mRNA expression of **(C)** interleukin 1 beta (*Il1b*) and **(D)** Tumor necrosis factor alpha (*Tnfa*) in mucosa from the small intestine. *n* = 15–17. Two-way ANOVA with assessment of simple main effects (treatment, no-LPS versus LPS; diet, WSD versus SC) and interaction effect (treatment × diet) with Tukey’s correction for multiple comparisons **(B–D)**. Data are presented as mean ± SEM. LPS, lipopolysaccharide; SC, standard chow; WSD, western-style diet.

Our first step was to evaluate tolerance and acceptance to oral LPS. Purified *E. coli* LPS was administered in drinking water (0.33 mg/mL; ~2 mg LPS/mouse/day) for 1, 4, or 8 days. Daily visual inspection and objective measurements of water intake, weight development and general physical wellbeing established no concerns regarding potential toxic effects of LPS. To validate whether orally ingested LPS reached the intestine, we quantified small intestinal luminal LPS content using mTLR4 transfected reporter cells (HEKBlue^™^ TLR4 cells). LPS levels were significantly elevated in the small intestine of LPS administered mice compared to non-LPS exposed control mice, and most likely reached a steady state LPS concentration around 4 days following LPS exposure (*p* = 0.001 and *p* < 0.001 for duodenum and ileum, respectively) (Figure 1A).

Next, we assessed the levels of biologically active LPS (i.e., LPS recognized by TLR4 on HEK-Blue^™^-4 cells) in the intestine of animals from the long-term oral LPS and WSD experiment. Thirty-five days of oral *E. coli* LPS administration led to significantly higher levels of bioactive luminal LPS in both duodenum (*p* = 0.015) and ileum (*p* = 0.009) compared to non-LPS exposed mice for both diets (Figure 1B).

### Western style diet (WSD) diet, but not oral LPS administration, led to a significant, but minor increase in IL-1β mRNA expression in small intestinal mucosa

Inflammatory cytokines are signaling proteins released from innate immune cells with key roles in regulating immune responses often elicited by microbial exposures. To address whether the elevated LPS levels in the ileum affected local inflammatory cytokine expression following a 5 week intervention with LPS administered in the drinking water fed western style diet (WSD) or a low-fat standard chow diet (SC). We measured the expression of TNFα and IL-1β in mucosal ileum tissue (32). Neither TNFα nor IL-1β were affected by LPS administration (Figures 1C,D). However, we detected a statistically significant increase in IL-1β gene expression in WSD-fed animals compared to SC-fed groups (*p* = 0.002; Figure 1C). TNFα was not expressed differently in animals fed WSD or SC (Figure 1D).

### A WSD promoted weight gain, but oral LPS administration had no additional effect on body weight development or food intake

**Figure 2:**
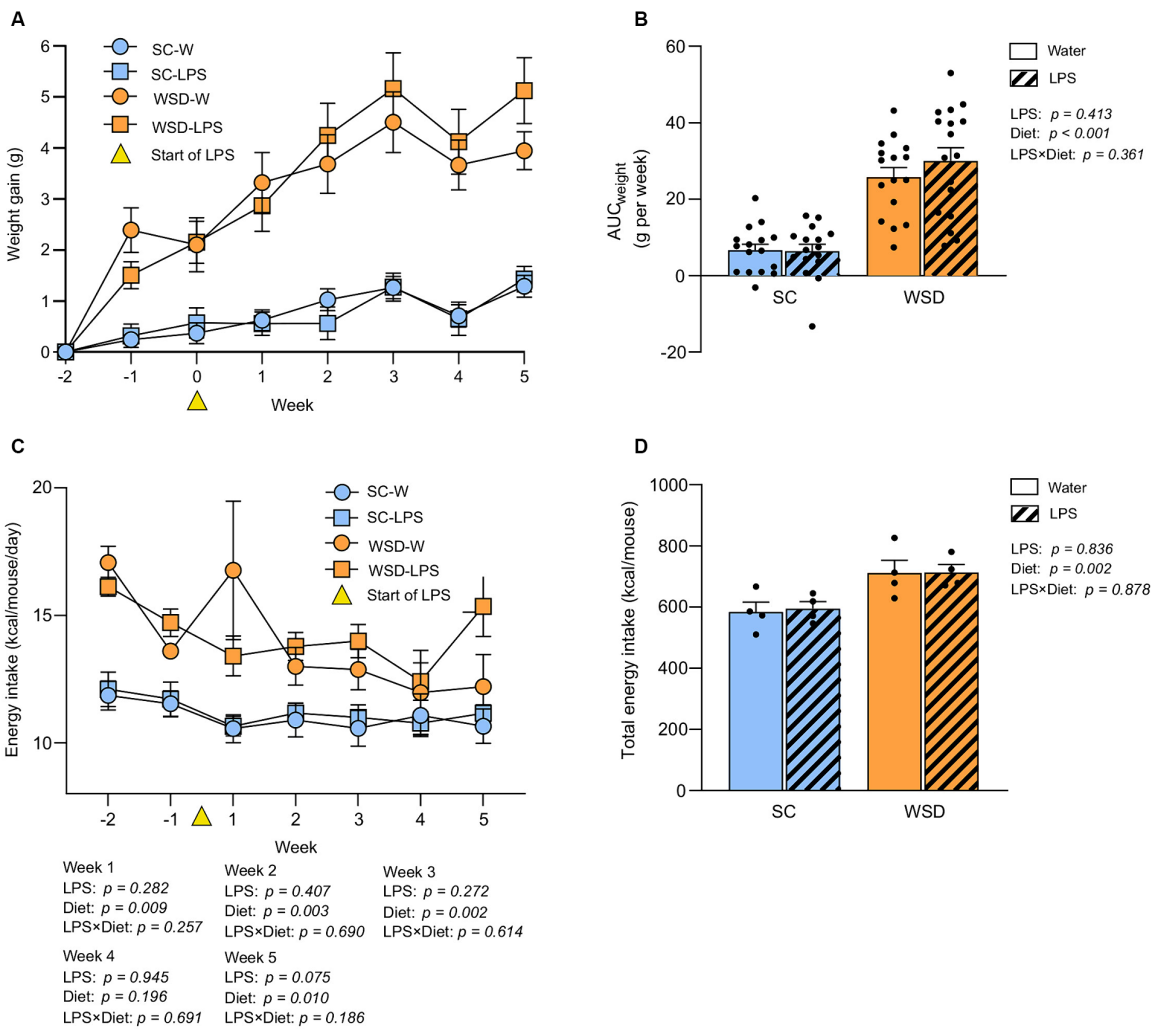
Weight and food intake assessed in mice fed WSD or SC diet for 7 weeks, and 35 days of oral LPS administration (0.33 mg/mL, estimated 2 mg LPS/mouse/day). **(A)** Body weight gain (G) of WSD or SC fed mice for 7 weeks. Week 0 (yellow triangle) indicates the start of LPS administration. **(B)** AUC body weight gain (g). **(C)** Weekly energy intake per diet group expressed as kcal/mouse/day. Week 0 (yellow triangle) indicates the start of LPS administration. **(D)** Total energy intake per diet group expressed as kcal/mouse. Two-way ANOVA with assessment of simple main effects (treatment, no-LPS versus LPS; diet, WSD versus SC) and interaction effect (treatment × diet) with Tukey’s correction for multiple comparisons **(B–D)**. Data are presented as mean ± SEM. *N* = 16–17 per group. AUC, area under the curve; LPS, lipopolysaccharide; ns, non-significant; SD, standard chow; WSD, western-style diet.

As expected, the WSD fed mice (WSD-W and WSD-LPS) gained significantly more weight than standard chow (SC) fed mice (SC-W and SC-LPS) during the 7 week dietary intervention (Figures 2A,B, *p* < 0.001). However, LPS administration did not affect weight gain (*p* = 0.413). Total body weight gain corresponded with total energy intake as the WSD fed mice had a higher energy intake than the SC mice (*p* = 0.002, Figure 2D), and administration of LPS had no significant effect on energy intake (*p* = 0.836, Figure 2D). Weekly energy intake of mice receiving WSD had the highest energy intake throughout the experiment regardless of LPS exposure, with a small exception at week 4 where no difference between groups was observed (Figure 2C).

### Whole-body glucose metabolism and intestinal IAP activity were not affected by oral LPS administration or WSD feeding

**Figure 3:**
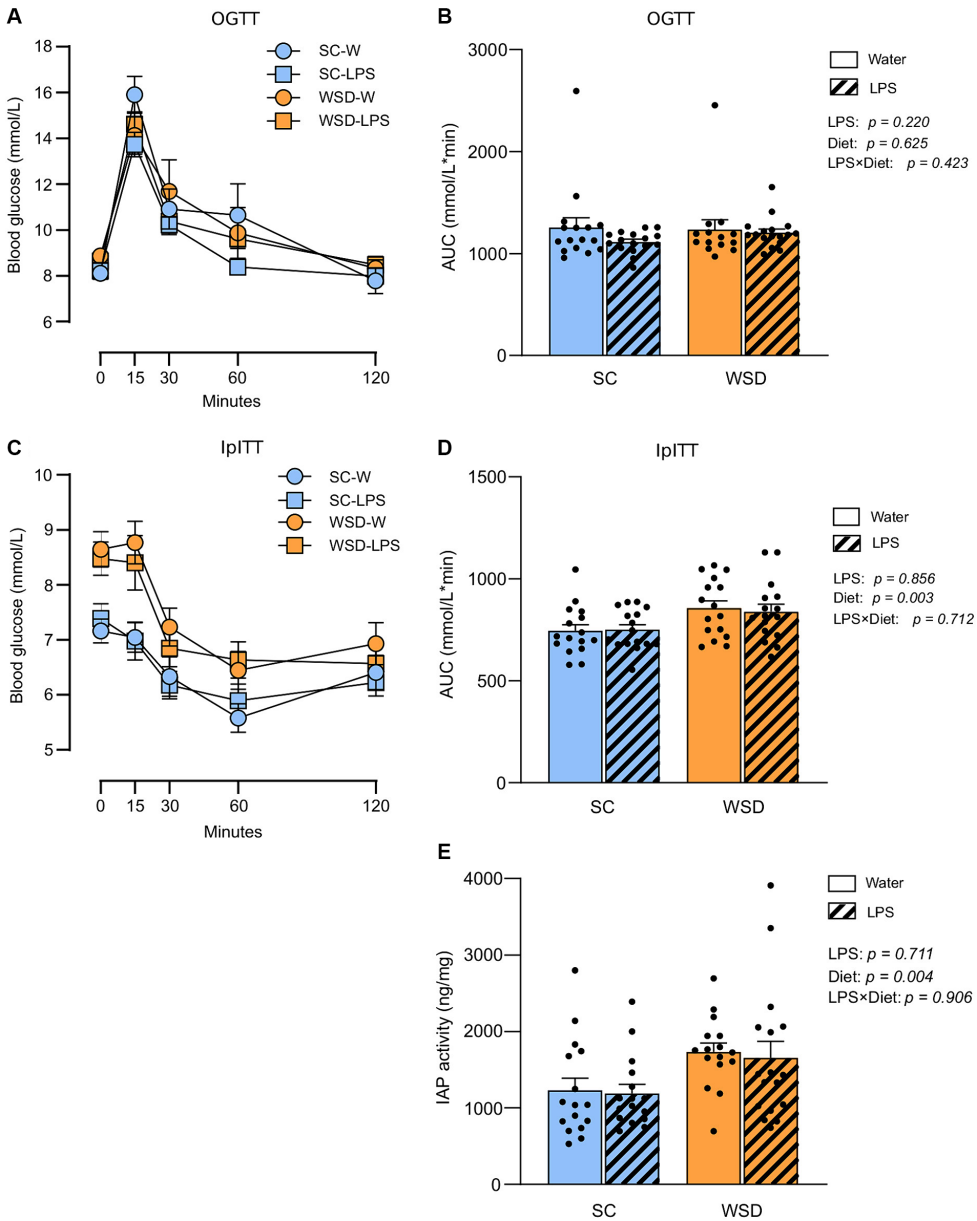
Oral glucose tolerance test (OGTT) and intraperitoneal insulin tolerance test (IpITT) in mice fed WSD or SC diet for 7 weeks, and 35 days of oral LPS administration (0.33 mg/mL, estimated 2 mg LPS/mouse/day). **(A)** OGTT. **(B)** Total glucose excursion after OGTT expressed as area under the curve. **(C)** IpITT. **(D)** AUC IpITT. **(E)** IAP activity from duodenal lumen content. Two-way ANOVA with assessment of simple main effects (treatment, no-LPS versus LPS; diet, WSD versus SC) and interaction effect (treatment × diet) with Tukey’s correction for multiple comparisons **(B,D,E)**. Data are presented as mean ± SEM. AUC, area under the curve; IAP, intestinal alkaline phosphatase; LPS, lipopolysaccharide; ns, non-significant, WSD, western-style diet.

We hypothesized that long-term exposure to orally administered LPS would elevate plasma LPS and induce insulin resistance as this would sufficiently challenge the tolerance of mice towards LPS. Accordingly, we evaluated the effect of chronic oral LPS administration on glycemic control and insulin resistance. We took advantage of the oral glucose tolerance test (OGTT), and the intraperitoneal insulin tolerance test (IpITT) to evaluate glucose regulation and whole-body insulin sensitivity, respectively, (Figures 3A–D). The OGGT showed no significant differences between different diets (*p* = 0.625) or treatment with LPS (*p* = 0.220). The effect of diet and LPS exposure collectively was also non-significant (*p* = 0.423; Figures 3A,B). In the IpITT the glucose curve (AUC) following insulin injection showed a clear effect of diet type (*p* = 0.003) where WSD mice displayed higher values than SC-fed mice, but with no effect of LPS exposure (*p* = 0.856) or any interacting effects between the two experimental factors (*p* = 0.712). The significant higher AUC values seen with WSD-feeding is likely attributed to the higher fasting glucose levels we observed in the WDS animals compared to SC-fed mice at the time point when doing the IpITT (*p* = 0.001, with one-way ANOVA).

Intestinal alkaline phosphatase (IAP) detoxifies LPS by removing phosphate groups from the lipid A moiety, thereby inhibiting LPS-TLR4 interaction (33). Earlier studies have described increased IAP activity in high-fat fed rodents (34–36), or found increased IAP activity to be correlated with elevated LPS luminal concentrations in the murine small intestine (37). We hypothesized that IAP secretion would rise with increasing luminal LPS levels (37, 38) and fat feeding (39). We found no effect of LPS treatment (*p* = 0.711), or any interaction effect between diet and LPS (*p* = 0.906) (Figure 3E). However, the WSD groups displayed statistically significant higher IAP activity than the low-fat SC groups (*p* = 0.004).

### NF-κB activity in liver- and abdominal region was transiently upregulated by oral LPS administration and WSD feeding

The transcription factor NF-κB is a primary regulator of inflammatory responses, and its activation is instrumental for the proinflammatory state associated with obesity and the detrimental effects on insulin resistance (2, 40). We hypothesized that mice fed the WSD would display increased global activation of the NF-κB signaling pathway, and that exposure to LPS would generate further increases in NF-κB activity. By exploiting a transgenic reporter model for NF-κB activity (NF-κB luc) (27), we assessed by *in vivo* imaging NF-κB activity in mice from all the experimental groups at a series of time points throughout the experiment.

**Figure 4:**
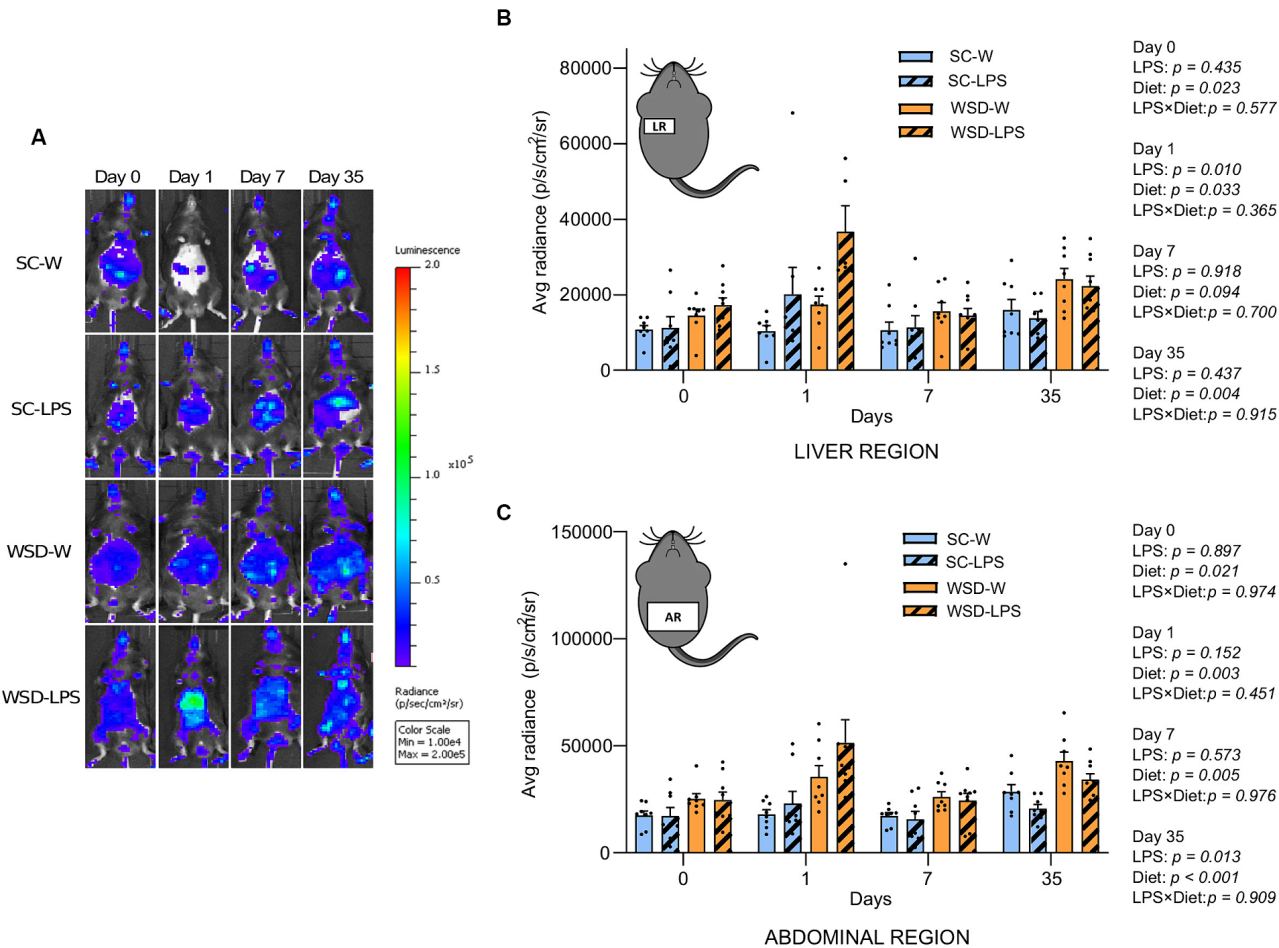
*In vivo* imaging of NF-κB activation during 35 days of LPS administration in drinking water (0.33 mg/mL, estimated 2 mg LPS/mouse/day). Day 0 indicates start of oral LPS administration Graphs display the photon emission in region of interest (ROIs) representing NF-κB activity in the liver region **(A)** and abdominal region **(B)** *N* = 8 per group. **(C)** Panel displays representative images of one mouse per group at different time points. Color bar indicates intensity of light emission from the mice. Two-way ANOVA with assessment of simple main effects (treatment, no-LPS versus LPS; diet, WSD versus SC) and interaction effect (treatment × diet) with Tukey’s correction for multiple comparisons **(B,C)**. Data are presented as mean ± SEM. LPS, lipopolysaccharide; NF-κB, nuclear factor-kappaB; SC-W, standard chow + water; SC-LPS, standard chow + LPS; WSD, western-style diet; WSD-W, western-style diet + water; WSD-LPS, western-style diet + LPS.

We observed a significant and transient increase in NF-κB activity from the liver in LPS administered mice 24 h after start of oral LPS administration compared to mice receiving only water (*p* = 0.010) (Figures 4A,B). Additionally, we found that WSD significantly led to a higher NF-κB activity in the liver at this time point (*p* = 0.033), but the analyses revealed that no interaction effect between diet and LPS (*p* = 0.365) was evident, meaning that LPS was not dependent on WSD for the increased liver NF-κB activity. When comparing each experimental group, only SC-W and WSD-LPS were significantly different (*p* = 0.006), however, the results showed a trend between the two WSD groups at day 1 (*p* = 0.059). After 1 week no differences between groups were observed. However, we observed again a significant increase in NF-κB activity in the WSD groups relative to SC-fed control mice at day 35 (*p* = 0.004). The abdominal region includes the intestines which is connected to the mesentery. The mesenteric tissue is rich in lymph nodes and functions as the “first pass” organ for microbial substances entering the lymph from the intestinal tract. This immediate exposure to substances coming from the intestinal lumen, such as LPS, may therefore trigger NF-κB activation in lymphatic immune cells. Our results showed that NF-κB activity was elevated in the abdominal region throughout the experimental period in mice fed WSD (Figure 4B), with no additional increase caused by LPS. Intriguingly, we observed on the contrary that LPS treatment reduced the abdominal NF-κB signal at day 35 (*p* = 0.013), causing speculations of a compensating effect against LPS exposure with time.

**Figure 5:**
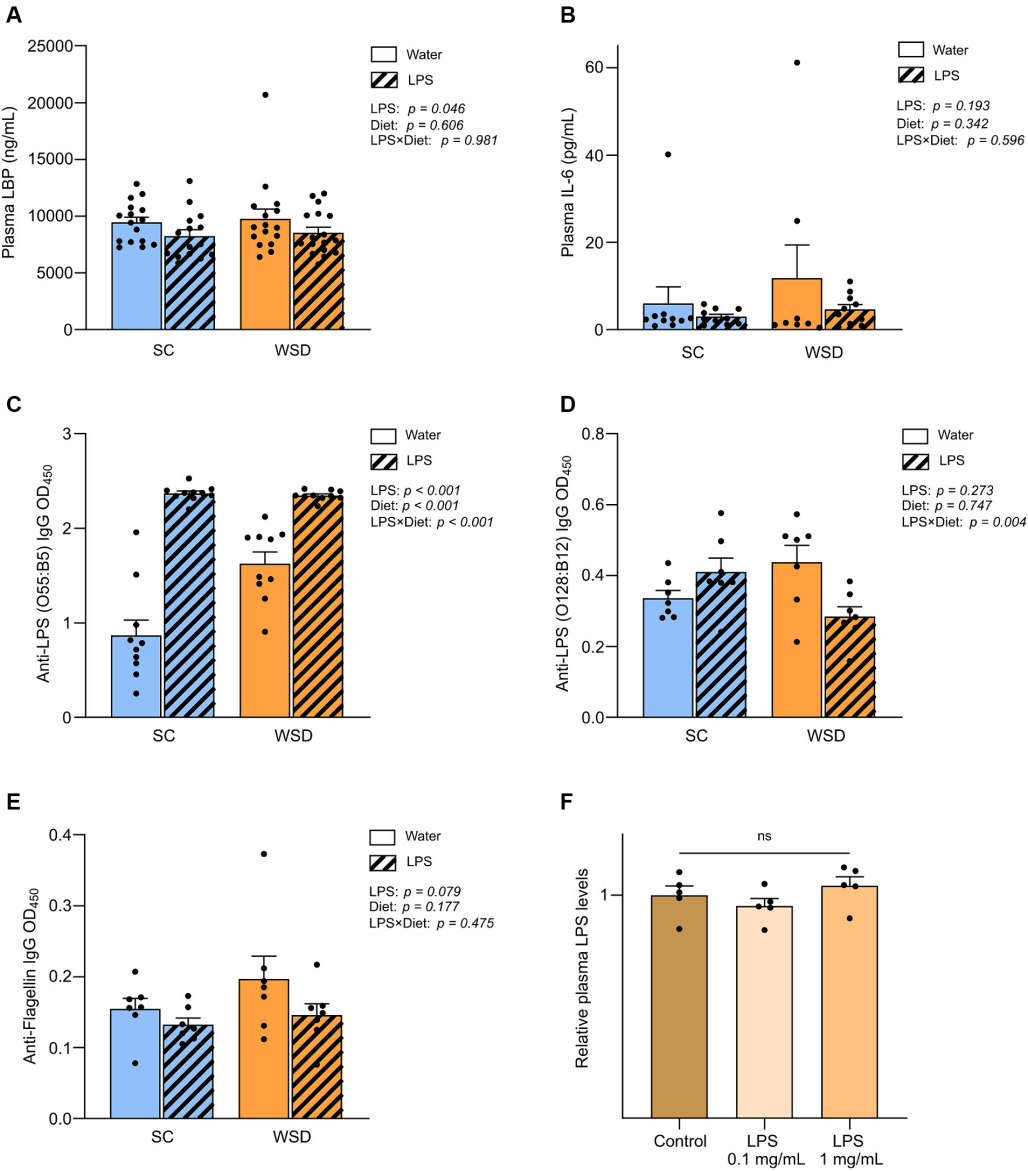
Plasma measurements of LBP, IL-6, immunoglobulins and LPS. **(A)** Measurement of plasma LBP, **(B)** IL-6, **(C)** Anti-LPS (O55:B5) IgG, **(D)** Anti-LPS IgG (O128:B12) and **(E)** Anti-flagellin IgG in mice fed WSD or chow diet for 7 weeks, and 35 days of oral LPS administration (0.33 mg/mL, estimated 2 mg LPS/mouse/day). Two-way ANOVA with assessment of simple main effects (treatment, no-LPS versus LPS; diet, WSD versus SC) and interaction effect (treatment × diet) with Tukey’s correction for multiple comparisons **(A–E)**. **(F)** Relative plasma LPS levels with normalized values from mice administered with oral LPS for 8 days compared to control group (water). One-way ANOVA compared to the mean of control group (non-treated mice). Data are presented as mean ± SEM. *N* = 16–17 (LBP), 7 (IL-6, anti-flagellin IgG and anti-LPS IgG), and 5 (LPS) per group. IgG, immunoglobulin G; LBP, LPS-binding protein; LPS, lipopolysaccharide; ns, non-significant; WSD, western-style diet.

As there are other inflammatory pathways independent of NF-κB activity, we included plasma IL-6 and TNFα measurements from day 35 to investigate if systemic inflammation was affected by the different treatments. Levels of plasma IL-6 were inside normal range for adult mice in all experimental groups (41, 42), and the results showed no significant differences between the groups (Figure 5B). Plasma TNFα levels were not possible to calculate as all samples ranged below detection limit.

### Oral LPS administration lowered lipopolysaccharide-binding protein levels in plasma, but LPS was not detected in circulation

Lipopolysaccharide-binding protein (LBP) is stable in blood and has a longer half-life in blood than LPS and is thus regarded as a useful biomarker of LPS exposure in circulation (43–45). Measurements of plasma LBP revealed firstly that the diet as such did not affect LBP levels (*p* = 0.606) (Figure 5A). However, in contrast to what we hypothesized exposure to LPS led to significantly lower levels of circulating LBP compared to non-LPS exposed mice (*p* = 0.046). To further explore the unexpected drop in LBP levels with LPS administration, we included a smaller follow-up experiment to measure LPS levels directly in plasma using a mTLR4-transfected cell-based colorimetric assay after 8 days of oral LPS administration (0.1 mg/mL and 1 mg/mL, estimated daily intake of 0.5 mg and 5 mg LPS, respectively). We were not able to quantify plasma LPS levels in any of the groups because values were below detection limit (0.001 ng/mL), indicating that LPS was either absent or bound up, making the LPS molecule unavailable for reacting with the TLR4-receptor. Relative comparison between group means showed that there were no significant differences in plasma LPS levels (*p* = 0.288) (Figure 5E).

### Plasma antibody levels against LPS (*E. coli* O55:B5) increased in oral LPS administered animals, but bacterial flagellin antibodies were not altered by oral LPS administration or WSD feeding

As the bacterial protein flagellin, and LPS, are usually excluded from absorption by the gut epithelium, a period of gut-barrier dysfunction translocation of LPS and flagellin across the intestinal mucosa may activate the adaptive immune response resulting in the generation of anti-flagellin and anti-LPS antibodies. As circulating levels of flagellin- and LPS specific immunoglobulin G (IgG) may serve as markers of long-term systemic exposure to flagellin and LPS, we sought to measure their presence in the plasma samples as an indirect measure of long-term gut leakage. Since we administered LPS derived from *E.coli*, serotype O55:B5, we specifically looked for antibodies raised against this LPS. In addition we assessed antibodies against a related LPS (*E.coli* serotype O128:B12) and flagellin. The latter two were chosen as representatives of a general gut leakage. Antibody levels against *E. coli* O55:B5 LPS were significantly and robustly increased in the LPS administered mice compared with control mice, *p* < 0.001 (Figure 5C). Interestingly, levels of antibodies against LPS serotype O128:B12 or flagellin were not significantly altered when comparing diet groups and/or LPS exposure (Figure 5E). Intriguingly, we observed a trend that anti-flagellin levels were in fact lower in LPS administered mice compared to controls (*p* = 0.079), in line with our observations on LBP-levels.

## Discussion

Consumption of high-fat, low-fiber, western-style diets (WSD) has been linked to obesity, low-grade inflammation, and poor glucose control, often attributed to the “leaky gut” hypothesis stating that intestinally derived bacteria or bacterial products can contribute to systemic inflammation (6, 46, 47). We expected that the higher content of dietary fat in the WSD would facilitate transportation of LPS over the intestinal wall and lead to systemic low-grade inflammation in mice administered LPS in the drinking water. We hypothesized that continuous oral LPS administration would further aggravate the proposed pro-inflammatory effect of the WSD, and even cause symptoms of metabolic disease in standard chow (SC)-fed mice. However, LPS supplementation did not assert any adverse consequences on glucose metabolism when testing for glucose intolerance and insulin resistance. Further, we did not detect strong indications of low-grade inflammation except for a transient upregulation of NF-κB activity in LPS administered mice during the first day of oral LPS exposure. These findings cohere with a comprehensive study in both mice and humans, which explored the impact of orally ingested LPS on intestinal barrier function and systemic inflammation (26). The authors found that chronic oral LPS intake promoted hepatic inflammation regardless of no substantial impact on energy intake, weight gain, intestinal inflammatory markers, intestinal barrier function, or glucose control assessed through HOMA-IR. Collectively, our results demonstrate that LPS situated in the intestine was not able to initiate systemic low-grade inflammation in animals receiving either WSD or SC.

There are a limited number of studies that address the direct link between small intestinal LPS concentrations and circulating LPS levels in models of high fat feeding. Exceptions are the forementioned study by Faraj et al. (26), and another study related to ours, where high fat fed mice were orally administered with *E. coli* strains that colonized the mouse gut and had differential expression of LPS (potent or weak). *E. coli* colonization led *per se* to increased inflammation in adipose tissue and dysregulated glucose control, but the latter effect was not dependent on LPS, which to a considerable extent corroborated with our findings (48). Yet, intestinal-derived LPS has been assigned a critical role in metabolic endotoxemia and low-grade inflammation (4, 6, 49, 50). Our measurements of LPS in luminal content from the small intestine showed that the number of TLR4-stimulants increased significantly in mice exposed to oral LPS obtained from *E. coli* both short term and long term, whereas non-exposed mice possessed only minor intestinal quantities of TLR4-stimulants. These findings may reflect that in our animals, most of the inherent LPS variants found in the intestinal lumen are not potent stimulators of TLR4. This observation is in line with other studies stating that the greater part of normal intestinal Gram-negative bacteria is of species from the bacteroidetes family that do not stimulate TLR4 signaling (24, 25). Hence, the ingested *E. coli*-derived LPS contributed to the elevated levels of TLR4-stimulatory environment of the small intestine in our experiment.

Although we observed large quantities of LPS in the small intestine, we could not through direct methods detect biologically active, TLR4-stimulating LPS in blood plasma. It should be noted that most studies supporting HFD-induced metabolic endotoxemia are based on LPS assessments by the limulus amebocyte lysate (LAL) assay (4, 6, 17). This method is dependent on the properties of the lipid A moiety and does not reflect the LPS’ ability to stimulate inflammation through the TLR4 signaling pathway, unlike the cell-based assays we have used. Consequently, the LAL test may overestimate the actual levels, and be misleading when correlating circulatory LPS levels and metabolic inflammation since the measured LPS is likely incapable of stimulating proinflammatory pathways in humans (51).

To further explore whether diet and LPS exposure would lead to other perturbations in the intestine we measured leakage of bacterial products into the circulation. In this context, we measured the plasma-levels of antibodies against LPS from serotype O55:B5, which was identical to the LPS administered in the water, and *E.coli* derived flagellin and LPS (O128:B12), the latter two representing general products from gut bacteria. Elevation in anti-flagellin and anti-LPS Ig concentrations have previously been observed in cases of chronic enteric conditions such as short-bowel syndrome (SBS) and inflammatory bowel diseases (IBD) (31, 52). Using flagellin- and LPS specific immunoglobulins as markers of long-term exposure to flagellin and LPS may therefore better capture long term gut leakage, than measuring either flagellin or LPS directly in blood as they can be more fluctuating (31). Antibodies against LPS O55:B5 was clearly higher in both mouse groups that had received LPS, suggesting that LPS given in the drinking water indeed has reached the circulation in sufficient quantities to raise an immune response against this particular LPS. However, the results also indicate that the presence of LPS in the circulation has later been neutralized and/or activated mechanisms of endotoxin tolerance. For flagellin and LPS (O128:B12) we see no increase in antibody-levels as a result of LPS administration. This indicates that LPS administration has not led to generally diminished intestinal barrier. On the contrary we observe a lower production of antibodies against flagellin in LPS administered mice. Collectively, our results imply that oral LPS exposure leads to only minor leakage into circulation of LPS and/or other bacterial products even with high-fat feeding, and that the animals quickly adapted to a higher LPS exposure, indicating acquired tolerance..

Supporting the notion that tolerance towards LPS was obtained is the unexpected results demonstrating reduced LBP levels in mice receiving oral LPS compared to controls not receiving LPS. Initially, this seemed contradictory as LPS promotes synthesis of LBP in liver and other tissues including the intestine (53–55). Therapeutic administration of high doses of LBP has also been shown to protect normal mice from LPS challenge (56). We can hypothesize that the low LBP levels are a consequence of acquired tolerance to chronic LPS administration, as previously demonstrated in a rat model of acute vs. chronic LPS exposure (57). Endotoxin tolerance is defined as the reduced capacity of a cell to respond to LPS after an initial exposure to this stimulus (58, 59). When the phenomenon endotoxin tolerance was introduced, the focus was largely on how treatments with low doses of LPS could prevent lethal outcomes of sepsis and fever. A broader understanding of endotoxin tolerance has been revealed through studies of possible beneficial mechanisms in areas of various chronic inflammatory diseases (60, 61) and other more specific areas such as cancer immunotherapy (58, 62). Endotoxin tolerance can be divided into early and late mechanisms of tolerance. Early tolerance is usually observed within hours of LPS administration and may last about a week. Early mechanisms involve altered cellular activation with suppression of cytokine production and fever independent of antibody production (63, 64). In contrast, late tolerance is induced many days after initiation of LPS treatment when early tolerance has generally ceased or declined. The molecular signature of endotoxin tolerance involves several mechanisms such as downregulation of TLR4 expression and decreased activation of intermediates in NF-κB signaling (58). Additionally, various negative regulatory molecules, for instance IL-1 receptor-associated kinase M (IRAK-M) and A20, are upregulated in cells tolerant to endotoxins and inhibit TLR signaling activation (58, 65, 66). Proposed mechanisms aside, exposure to immunostimulatory LPS has been shown to contribute to protection from immune-mediated diseases by modulating the immune system responsiveness (25). With the “hygiene hypothesis” in mind, early life exposure to potent immune activating LPS led through endotoxin tolerance to immune education and better protection against autoimmune disease than exposure to less inflammatory subtypes of LPS.

Had LBP concentrations been measured right after start of LPS exposure, we may assume that LBP levels would have been elevated in this initial phase as a protective mechanism after acute stress such as LPS challenge (53), before decreasing as a result of LPS tolerance. Moreover, our results demonstrated that plasma LBP levels were entirely independent on the assigned diet type and the final body weight of the mice and contrasts with many other studies demonstrating that high fat diets increase LBP levels in blood (16, 37). However, in line with our observations, a human intervention study showed that LBP levels were not elevated despite significant weight gain following a high-fat diet (11).

With regards to duodenal IAP, we initially hypothesized that this enzyme would have increased activity after oral LPS exposure to protect against toxic LPS activity (38, 67, 68). Surprisingly, IAP activity in duodenum was not affected by chronic LPS exposure despite elevated levels of LPS in the small intestine relative to non-LPS exposed animals. We instead observed a significant increase in small intestinal IAP activity in non-LPS exposed WSD-fed mice relative to SC-fed mice. Previous studies have shown that IAP regulates intestinal lipid absorption (34, 69, 70), and reduces weight gain in mice fed a high-fat diet (69). Moreover, IAP expression is increased in high fat diet fed mice, but it appears in some studies that this adaptive mechanism is not sufficient to counteract dietary fat stimulated LPS translocation from the intestine (33, 70).

NF-κB is a family of transcription factors instrumental in orchestrating the inflammatory response and is highly sensitive to LPS. We observed a temporary, inflammatory induction of NF-κB activation in the liver of mice receiving WSD-LPS diet after only one day of the feeding period. The transgenic NF-κB mice used in this study are sensitive to even minute changes in LPS quantity and can detect LPS levels in the range of 0.1–0.4 ng/mL using bioluminescent imaging tools (unpublished). This indicates that, if the orally derived LPS reached the bloodstream, the levels are below 0.1 ng/mL at the 5 week time point, which corresponds with our negative finding when measuring LPS directly in plasma with the HEK-Blue assay (detection limit 0.001 ng/mL). We can only speculate whether the upturn in NF-κB activation in both WSD-groups at the later stage of the experimental period might be indicative of an initiating, but not yet systemic, low-grade inflammation. This hypothesis is partly supported by another study in C57BL/6 mice which showed that the inflammatory response to a HF diet developed in two distinct phases; one immediate after a few days concurrent with an acute phase response, and a second increase after 12–16 weeks that correlated with WAT and muscle inflammation (71). The authors proposed that the initial response resulted from an acute and transient inflammation and activation of liver cells, which fits with our findings. As there are other inflammatory pathways independent of NF-κB activation, we analyzed plasma levels of two central proinflammatory cytokines: TNFα and IL-6. The results from these analyses supported our other findings that we could not find evidence of an ongoing low-grade systemic inflammation.

The results from our experiments point to that exposure to ingested LPS, high-fat diet or not, does not impair the intestinal barrier or trigger the development of chronic low-grade systemic inflammation. We wanted to lessen the possibility of missing whether our experimental factors could initiate a local intestinal inflammation that at a later stage could develop into a low-grade systemic inflammation. To do so we aimed to measure the state of intestinal inflammation through gene expression analyses of proinflammatory cytokines in the small intestinal mucosa. Others have found in murine models of intestinal inflammation, that TNFα and IL-1β appear to be protective during acute inflammation but exert pure proinflammatory functions in later stages of inflammation (32). In addition to studies researching the role of high fat diets and LPS in situations it is plausible that in subjects with weakened barriers (e.g., inflammatory bowel diseases such as Crohn’s disease or ulcerative colitis), leakage of LPS or other bacterial products from the intestine can have significant effects on systemic of low-grade systemic inflammation (see Introduction), both exposure to a western-style diet (72, 73) and intestinal LPS (74, 75) have previously been shown to increase intestinal inflammation through various possible mechanisms. When evaluating the local inflammatory status in small intestinal mucosa, we did not find any indication that WSD-feeding or oral exposure to LPS caused changes in proinflammatory factors of the intestinal immune defense.

Our results do not dismiss that LPS enter the circulation from the intestine, but the results point to the fact that the animals’ defense system managed the intruding LPS well and prevented, or slowed, the development of low-grade systemic inflammation. It is important to remember that these mice were metabolically healthy, and most likely had a robust intestinal barrier. The metabolic syndrome and associated diseases are characterized by a state of dyslipidemia. Recent studies have proposed that LPS is an relevant regulator of areas in lipid metabolism such as modifying plasma lipid levels (76) and activating cholesterol metabolism processes (77), further connecting LPS to dyslipidemia and systemic inflammation. Even though our results point to little or no metabolic dysregulation, a limitation of this study is that we did not explore LPS’ effect on lipid metabolism or markers of sustained inflammation such as gene expression in the liver or relevant biomarkers in plasma. It has been suggested that other factors in the modern lifestyle, such as exposure of processed food rich in certain food additives (e.g., emulsifiers), can lead to “leaky guts” and contribute to lifestyle diseases such as cardiovascular disease, cancer, and type 2 diabetes (30, 78). Hence, an uncontrolled increase in intestinal permeability to bacterial or other foreign molecules can play a significant role in disease development when consuming an unhealthy diet for an extended time. However, the present study creates new insights into mucosal tolerance levels to proinflammatory LPS in concert with dietary fat exposure, suggesting that LPS residing in the intestine might be less harmful than proclaimed. Further studies are now necessary to better understand the mechanisms behind restored immune responsiveness after LPS exposure.

## Data availability statement

The original contributions presented in the study are included in the article/Supplementary material, further inquiries can be directed to the corresponding author.

## Ethics statement

The animal study was approved by Norwegian Food Safety Authority. The study was conducted in accordance with the Norwegian Regulation concerning the use of animals for scientific purposes and EU Directive 2010/63.

## Author contributions

SH: Formal analysis, Methodology, Project administration, Writing – original draft, Writing – review & editing, Investigation. VS: Writing – review & editing, Methodology, Investigation. BE: Supervision, Writing – review & editing, Funding acquisition, Project administration. JP: Writing – review & editing. HC: Conceptualization, Formal analysis, Methodology, Project administration, Supervision, Writing – original draft, Writing – review & editing, Investigation. SB: Writing – review & editing, Investigation, Formal analysis, Methodology.

## Glossary

CD14: cluster of differentiation 14
DIO: diet-induced obesity
*E. coli*: *Escherichia coli*
ELISA: enzyme-linked immunosorbent assay
GAPDH: glyceraldehyde-3-phosphate dehydrogenase
GIT: gastrointestinal tract
HEK: human embryonic kidney
HOMA-IR: homeostatic model assessment for insulin resistance
IAP: intestinal alkaline phosphatase
IgG: immunoglobulin G
IL-1β: interleukin-1 beta
IL-6: interleukin-6
IpITT: intraperitoneal insulin tolerance test
LAL: limulus amebocyte lysate
LBP: LPS-binding protein
LPS: lipopolysaccharides
MD-2: myeloid differentiation factor 2
NF-κB: nuclear factor-kappaB
NF-κB-luc: nuclear factor-κB-luciferase
OD: optical density
OGTT: oral glucose tolerance test
SC: standard chow
RT-qPCR: reverse transcription-quantitative polymerase chain reaction
TLR4: toll-like receptor 4
TNFα: tumor necrosis factor alpha
WAT: white adipose tissue
WSD: western-style diet
WT: wild type
