# Supplementary material for: Chronic oral LPS administration does not increase inflammation or induce metabolic dysregulation in mice fed a western-style diet
Source: Front Nutr. 2024 Jul 15;11:1376493. doi: 10.3389/fnut.2024.1376493 (PMC11284168; doi:10.3389/fnut.2024.1376493)
Supplement: Supplementary file 1 [file Table_1.DOCX]

**Supplementary tables**

**Table S1.** The dietary composition of macronutrients and energy values of the diets used in this study.

| **Dietary Composition** |  | | **Standard Chow**  **(SDS RM1)** | **Western-style Diet**  **(RD D12079B w/**  **70 g cellulose)** |
| --- | --- | --- | --- | --- |
| Percentage weight: | Carbohydrate (digestible) |  | 66.1 | 49.2 |
|  | (% of total CHO) | Starch | 68.0 | 8.9 |
|  |  | Cellulose | 6.5 | 12.5 |
|  |  | Fiber, other | 19.4 | - |
|  |  | Maltodextrin | - | 17.8 |
|  |  | Sugar | 6.1 | 60.8 |
|  | Protein |  | 14.4 | 20.0 |
|  | Fat |  | 2.7 | 21.0 |
|  | (% added to feed) | Cholesterol | - | 0.15 |
|  | (% of total fat) | SFA | 22.4 | 62.4 |
|  |  | MUFA | 38.8 | 30.7 |
|  |  | PUFA | 38.8 | 13.6 |
|  |  | Total | 100 | 100 |
| Percentage energy: | Carbohydrate |  | 75.1 | 43.0 |
|  | Protein |  | 17.5 | 17.0 |
|  | Fat |  | 7.4 | 40.0 |
|  | Total |  | 100 | 100 |
| Total energy (kcal/kg): |  | | 3 520 | 4 686 |
| CHO, carbohydrates; MUFA, monounsaturated fatty acids; PUFA, polyunsaturated fatty acids; SFA, saturated fatty acids. | | | | |

**Table S2.** Fatty acid profile of Western-style diet (RD D12079B)

| **Fat source** | **Milk/Corn oil** |
| --- | --- |
| **Ingredients** | **g** |
| Butter, Anhydrous | 200 |
| Corn Oil | 10 |
| Total | 210 |
|  |  |
| **Fatty Acid** | **g** |
| C4, Butyric | 6.4 |
| C6, Caproic | 3.8 |
| C8, Caprylic | 2.2 |
| C10, Capric | 5.0 |
| C12, Lauric | 5.6 |
| C14, Myristic | 20.0 |
| C14:1, Myristoleic | 3.0 |
| C15, Pentadecanoic | 0.0 |
| C16, Palmitic | 53.5 |
| C16:1, Palmitoleic | 4.6 |
| C17, Heptadecanoic | 0.0 |
| C18, Stearic | 24.4 |
| C18:1, Oleic | 52.7 |
| C18:2, Linoleic | 10.6 |
| C18:3, Linolenic | 2.9 |
| C20, Arachidic | 1.9 |
| C20:1, Eicosenoic | 0.0 |
| C20:2, Eicosadienoic | 0.0 |
| C20:3, Dihomo-gamma-linolenic | 0.0 |
| C20:4, Arachidonic | 0.0 |
| C22:5, Docosapentaenoic | 0.0 |
| **Total** | 196.6 |
| Saturated | 122.8 |
| Monounsaturated | 60.3 |
| Polyunsaturated | 13.6 |
|  |  |
|  | **% (wt: wt)** |
| Saturated | 62.4 |
| Monounsaturated | 30.7 |
| Polyunsaturated | 6.9 |

Wt, weight.

**Table S3.** Primer pair sequences for qPCR

| **Primer pair** | **Sequence*** | **Melting temperature (°C)** | **Amplicon (bp)** |
| --- | --- | --- | --- |
| *TNFa* | F: CTGTCTACTGAACTTCGGGGTGAT  R: GGTCTGGGCCATAGAACTGATG | 61 | 88 |
| *IL1b* | F: GCAGCTGGAGAGTGTGGAT  R: AAACTCCACTTTGCTCTTGACTT | 61 | 97 |
| *Gapdh* | F: CTTCAACAGCAACTCCCACTCTT  R: GCCGTATTCATTGTCATACCAGG | 60 | 103 |
| *Forward (F) and reverse (R) primer DNA 5’-3’ sequence | | | |
